# Transformers for Multi-Horizon Forecasting in an Industry 4.0 Use Case

**DOI:** 10.3390/s23073516

**Published:** 2023-03-27

**Authors:** Stanislav Vakaruk, Amit Karamchandani, Jesús Enrique Sierra-García, Alberto Mozo, Sandra Gómez-Canaval, Antonio Pastor

**Affiliations:** 1 Departamento de Sistemas Informáticos, Escuela Técnica Superior de Sistemas Informáticos, Universidad Politécnica de Madrid, 28031 Madrid, Spain; stanislav.vakaruk@upm.es (S.V.); amit.kbatra@alumnos.upm.es (A.K.); sm.gomez@upm.es (S.G.-C.); antonio.pastorperales@telefonica.com (A.P.); 2 Departamento de Ingeniería Electromecánica, Escuela Politécnica Superior, Universidad de Burgos, 09006 Burgos, Spain; jesierra@ubu.es; 3 Telefónica I+D., 28050 Madrid, Spain

**Keywords:** deep learning, time series, multi-horizon forecasting, transformer, multi-access edge computing, Industry 4.0, automated guided vehicles, 5G

## Abstract

Recently, a novel approach in the field of Industry 4.0 factory operations was proposed for a new generation of automated guided vehicles (AGVs) that are connected to a virtualized programmable logic controller (PLC) via a 5G multi-access edge-computing (MEC) platform to enable remote control. However, this approach faces a critical challenge as the 5G network may encounter communication disruptions that can lead to AGV deviations and, with this, potential safety risks and workplace issues. To mitigate this problem, several works have proposed the use of fixed-horizon forecasting techniques based on deep-learning models that can anticipate AGV trajectory deviations and take corrective maneuvers accordingly. However, these methods have limited prediction flexibility for the AGV operator and are not robust against network instability. To address this limitation, this study proposes a novel approach based on multi-horizon forecasting techniques to predict the deviation of remotely controlled AGVs. As its primary contribution, the work presents two new versions of the state-of-the-art transformer architecture that are well-suited to the multi-horizon prediction problem. We conduct a comprehensive comparison between the proposed models and traditional deep-learning models, such as the long short-term memory (LSTM) neural network, to evaluate the performance and capabilities of the proposed models in relation to traditional deep-learning architectures. The results indicate that (i) the transformer-based models outperform LSTM in both multi-horizon and fixed-horizon scenarios, (ii) the prediction accuracy at a specific time-step of the best multi-horizon forecasting model is very close to that obtained by the best fixed-horizon forecasting model at the same step, (iii) models that use a time-sequence structure in their inputs tend to perform better in multi-horizon scenarios compared to their fixed horizon counterparts and other multi-horizon models that do not consider a time topology in their inputs, and (iv) our experiments showed that the proposed models can perform inference within the required time constraints for real-time decision making.

## 1. Introduction

In recent years, the use of deep learning has gained widespread popularity for forecasting tasks due to its capacity to model complex non-linear relationships in time series [1,2]. With the ever-increasing availability of data and computational resources, deep-learning models have surpassed traditional methods, such as ARIMA, in terms of their prediction accuracy and robustness [3].

Today, long short-term memory networks (LSTM) are widely used for time-series prediction and have been shown to be highly effective in various domains, including economics [4], energy [5,6], and robotics [7]. The advent of transformers [8] has ushered in a new era in the field of artificial intelligence, attracting substantial attention in various research domains, most notably in natural language processing (NLP) and computer vision and also in disciplines requiring intricate sequence analysis, such as time-series prediction [9]. The innovative attention mechanism incorporated in this architecture has emerged as the fundamental concept that distinguishes this proposal from previous deep-learning algorithm designs.

A simplification of the original transformer architecture, called the encoder-only model was recently proposed in [10] to accommodate time-series-forecasting tasks. This proposal provided a source of inspiration for the two new variations of the transformer architecture that we propose in this work.

In mobile robotics, deep learning can be exploited to forecast robot performance and failures [7]. Among these mobile robots, automated guided vehicles (AGVs) stand out for their efficiency and versatility in industrial applications. These are automatic transport vehicles commonly used to replace conveyors and manual industrial trucks in the industrial sector [11]. They are widely applied to transport parts and assets in industrial manufacturing processes and automotive assembly plants, among others. The ability to control these AGVs remotely has increased the flexibility and safety of the logistic solutions, thus, allowing operators to manage multiple vehicles simultaneously in a centralized and reactive manner [12]. However, to make it possible, a reliable communication system with minimum latency for multiple AGVs is required.

5G mobile networks, due to their ultra-low latency (down to 1 ms) and high bandwidth, offer the right technology for these applications. In addition, 5G services enable the placement of edge-computing system that opens up the possibility of new services for industrial environments [13]. Thus, 5G networks, together with edge-computing services, allow simultaneous control of multiple AGVs with ultra-low latency. This scenario can be applied to virtualize and run in the edge the PLC (programmable logic controller) of an AGV, which can favor energy savings and scalability as PLCs can be created or stopped at demand in real time by simply running PLC software in a virtual machine in the edge of the 5G network.

However, although 5G mobile networks are considered to be more secure than previous generations [14], this does not imply a complete exclusion of potential communication problems. Indeed, in these remote-controlled applications, any network incident can produce downtime in the industrial process, which may result in serious economic losses [15]. Therefore, industrial risk prevention methods, such as those provided by deep-learning techniques, must be considered.

To contribute to the viability of these remote-controlled applications, in this work, we propose advanced forecasting methods to anticipate the deviation of the AGV trajectory in order to be able to take some corrective action (e.g., stop the AGV) that avoids harmful situations, such as an uncontrolled AGV running out of its trajectory and crashing against an object or person. In particular, we want to forecast the future state of an industrial AGV remote-controlled in a 5G scenario in which different types of network perturbations appear. Specifically, we focused on the prediction ahead of time of the AGV deviation from its defined trajectory.

This prediction provides valuable information about the quality of the navigation and, crucially, allows the implementation of preventive control strategies. For instance, the AGV can reduce its speed considering the predicted information so that the AGV can operate within preventive safety margins. To this end, we propose, for the first time, to adapt a transformer architecture, which is considered to be the state-of-the-art of neural network architectures, to the specificity of the forecasting problem that we want to solve.

To evaluate the advantage of adapting a transformer architecture to this forecasting problem, we compare the results obtained with our modified transformer architecture to those obtained using LSTM architectures, which are a type of typical deep-learning architecture used for forecasting applications.

In a previous work [15], it was shown how the use of traditional deep-learning models for fixed-horizon prediction can help to prevent AGV malfunctioning in a 5G scenario with network perturbations. In sharp contrast, in this work, we deeply analyze the advantages of multi-horizon forecasting paradigms against the fixed-horizon approach. To this end, we compare the performance provided by well-established deep-learning techniques, such as LSTM, to a multi-horizon model based on transformers, the cutting-edge architecture that has recently evolved the AI landscape and in particular the natural language processing (NLP) and large language model areas.

We adapted the transformer architecture in its encoder-only version [10] into a multi-horizon forecasting architecture with two different variants: (i) one that applies attention to all input data by flattening the input dimensions into one (we called this the TRA-FLAT architecture) and (ii) the other that applies attention solely on the time dimension (denoted as the TRA-TIME architecture). These two models were trained with data obtained from a real industrial AGV working in a remote-controlled 5G scenario subjected to different delay and packet-loss network disturbances. The details of the setup of this realistic 5G scenario were previously described in [15].

After running the designed experiments, the results obtained demonstrate that multi-horizon models provide a more flexible and robust forecasting solution compared with fixed horizon models as their performance is very similar to those of fixed horizon models in terms of accuracy; however, in addition, they provide a sequence of future predictions at different instants of time instead of a single point prediction in the future.

Additionally, we observed that the architectures that use inputs with temporal structure (e.g., time series) advantageously exploit this topological information and perform better in multi-horizon problems than in fixed-horizon problems. Furthermore, the proposed multi-horizon models were proven to be more robust and efficient for practical use in real-time industrial applications.

### 1.1. Contributions

The primary contribution of this research lies in the adaptation of the state-of-the-art transformer deep-learning architecture to a novel use case of multi-horizon forecasting within the context of Industry 4.0 scenarios. Specifically, we adapted the encoder-only transformer model in various ways to generate multi-horizon predictions. Our novel models represent a significant advancement compared to prior work in this area, which has primarily relied on fixed-horizon forecasting using LSTM and 1D-CNN deep-learning models. From this contribution, we derive the following secondary contributions:

- Innovative Transformer-Based Architectures for Multi-Horizon Forecasting: Two innovative variations of the encoder-only transformer architecture were devised: one that incorporates attention mechanisms applied to all input data (TRA-FLAT) and the other that applies attention solely to the time dimension (TRA-TIME). To the best of our knowledge, this represents the first attempt to adapt and apply an encoder-only transformer model to a multi-horizon forecasting problem. Our experimental results indicate that both transformer-based models outperform traditional deep-learning models (LSTMs) in terms of accuracy in both multi-horizon and fixed-horizon scenarios.
- Superiority of Temporal-Based Deep-Learning Architectures in Multi-Horizon Forecasting: This study presents empirical evidence in favor of deep-learning architectures that take advantage of temporal relationships in the data—specifically TRA-TIME and LSTM—for multi-horizon forecasting problems. Despite the conventional assumption that fixed-horizon models, which are optimized to predict a specific point in the forecasting horizon, would outperform their multi-horizon counterparts, our experiments indicate that architectures capable of exploiting the temporal structure of the data, given an adequate amount of past temporal data as input, achieve superior performance. Moreover, our findings suggest that TRA-TIME and LSTM outperform other multi-horizon models, such as TRA-FLA, that do not consider the temporal topology of the input data in multi-horizon forecasting scenarios.
- Advantages of Multi-Horizon Forecasting Models for Real-Time Forecasting: Our study suggests that, in the context of real-time forecasting scenarios, multi-horizon models represent a more efficient alternative to fixed-horizon models. This is attributed to the fact that a single multi-horizon model is capable of generating a sequence of forecasts across a given temporal range, as opposed to a single prediction for a particular time instant. Furthermore, the training and validation of multiple fixed-horizon models that span the same forecasting horizon requires more computational resources than required for a single multi-horizon model with equivalent precision. This finding is especially relevant in the context of Industry 4.0 real-time deployments, where periodic model retraining is necessary to address data drift issues throughout the lifespan of the deployed models.

### 1.2. Paper Structure

The remainder of the manuscript is organized as follows. In Section 2, related work is discussed. In Section 3, we introduce the selected deep-learning architectures, including the new transformer models proposed in this article. In Section 4, we describe the architecture of the system model. Section 5 depicts the dataset and the data collection process. We explain the use case infrastructure and the various network degradation scenarios that were run to collect the data. The data cleaning, processing, and separation into training/testing sets are also detailed in this section. In Section 6, the model training and evaluation procedures are described.

Section 7 details the results obtained from the experiments conducted. We first show that a multi-horizon forecasting model is superior to a fixed-horizon model. Then, we demonstrate that the deep-learning models with a sequence-related architecture outperform in multi-horizon forecasting problems. Finally, we demonstrate that the selected models are suitable for deployment in a real-time solution in the context of Industry 4.0. Section 8 provides a comprehensive summary of the key findings that were obtained from the conducted experiments. We conclude in Section 9 by summarizing the main conclusions derived from the results obtained and present interesting future work to be explored.

## 2. Related Work

In recent years, advances in AGV technology have enabled more flexible and efficient operations in a variety of applications. However, in the real world, AGVs must interact effectively with other automated systems and people, requiring proper management to avoid disruptions and ensure safe operation.

In practice, free movement of AGVs is not a viable option because it requires extensive and costly facility mapping. To overcome this limitation, researchers have proposed using guidelines to create a pre-defined route for AGVs. These guidelines can be in the form of physical markers, such as tapes or landmarks placed on the ground, or virtual routes stored in the vehicle’s memory. These lines are used to limit the movements of the AGVs and determine their position in the factory.

To realize the remote capability of the AGV, it is important to consider possible deviations from the predefined route as, otherwise, the AGV will not be able to navigate safely through the facility, leading to unexpected collisions with the environment and disruptions in the operational process. Time-series forecasting techniques have the potential to predict the future movement of AGVs and anticipate deviations from the predefined route.

In recent years, there has been growing interest in the application of DL models to time-series forecasting due to their ability to learn complex patterns in the data. In this regard, multiple deep-learning architectures have emerged that have exceeded LSTMs in multiple domains, such as those based on the transformer architecture [8].

Transformer-based architectures emerged in the field of natural language processing, where they are still considered state-of-the-art for sequence-to-sequence tasks. Several authors are working on adapting this architecture to the field of time series [9] and to regression problems by eliminating their “decoder” part, thereby, turning them into an encoder-only model [10]. To our knowledge, only two studies have been published to date that deal with a scenario in which an automated guided vehicle (AGV) is controlled remotely through a 5G network.

The authors of [16] introduced an AGV that is operated remotely using 5G equipment installed on the customer’s premises. They presented a simple control scheme for a single AGV that is automated through control commands that are sent from the 5G base station, based on visual information gathered by a camera mounted on the AGV and transmitted to the remote MEC platform via the 5G radio access network (RAN) link.

However, as the authors indicated, the control algorithms are founded on a basic kinematic model, lacking the use of any machine-learning or deep-learning techniques for anticipating the AGV’s trajectory. In contrast, our solution is based on predicting the deviations of the AGV’s trajectory in advance, which is of paramount importance to mitigate potential harmful situations that may arise from deviations caused by faults in the guidance control.

Another important drawback of the work presented in [16] is that it was not tested under a variety of realistic conditions in a real industrial environment, and therefore no evaluation was performed considering the effects of network disturbances and varying traffic loads in the remote control system. In comparison, our work evaluates the performance of deep-learning models in a real-world scenario where a wide range of network disturbances (e.g., delay and jitter) were simulated during AGV operation.

Moreover, the work in [16] is not based on a real-world Industry 4.0 setup, such as the one we propose, which uses industrial-grade components (programmable logic controller (PLC) and AGV) in the experiments. Finally, their work does not provide an analysis of the response time of the control algorithm, a crucial aspect to validate the feasibility of its deployment, which we address in our work.

In the work presented in [15], the use case used in this work was initially introduced with a proposed solution that focused mainly on demonstrating the ability to predict AGV malfunctioning in an industrial grade environment. This was achieved exclusively by analyzing the AGV-PLC connection without deploying any meter on the end-user equipment (AGV and PLC). However, our work extends this by focusing on evaluating the performance of the latest deep-learning techniques for multi-horizon prediction of remotely controlled AGV deviation in 5G networks affected by network degradation effects, and introducing two new and distinct versions of the transformer architecture for multi-horizon prediction that are not previously reported in the literature.

Furthermore, there are several important differences between our work and that of [15]. First, the solution proposed in our work involves predicting a sequence of 200 future values (from 0.1 to 20 s ahead of the instant time with steps of 100 ms) using powerful sequence-to-sequence DL models, rather than the single instantaneous value (the mean value between 10 and 15 s ahead of time) predicted using a typical regression strategy in [15]. Our approach allows for multiple horizons within the same model structure, providing the AGV operator with the flexibility to choose the most appropriate horizon for predicting the future deviation sequence based on current application needs without requiring model retraining and without compromising performance.

Second, the forecast horizon was extended from 15 to 20 s in our work, allowing for a greater safety margin to apply appropriate maneuvers to prevent the AGV from colliding with surrounding obstacles, thus, improving the safety of the work area.

Third, our work provides a comprehensive comparison of two new and different versions of the transformer architecture that are not previously reported in the literature, with traditional ML/DL models for comparative purposes, while in [15], only traditional DL algorithms (LSTM and 1D-CNN) were used.

Our work leverages the transformer architecture, for which two new and different variations of the encoder-only transformer architecture were proposed for the task of multi-horizon forecasting, viz. TRA-FLAT and TRA-TIME. The former model attended to all available features by flattening the input dimensions into a single vector, while the latter only considered the temporal features. We demonstrate that forecasting using the transformer architecture outperforms traditional deep-learning models for the task at hand. Moreover, we show that employing time-related multi-horizon predictors can significantly improve the robustness of the predictions.

Fourth, in sharp contrast to [15], which exclusively evaluated fixed-horizon predictors, this study examines the relative efficacy of both fixed-horizon and multi-horizon prediction models to predict the sequence of future deviations of the AGV. To accomplish this, we leverage state-of-the-art deep-learning architectures and adapt them for the task of multi-horizon time-series forecasting. After exploring a comprehensive range of hyperparameters and model combinations, our results suggest that multi-horizon predictors can be utilized with negligible loss of precision, enabling AGV operators to choose an appropriate forecasting step according to the factory’s workload, network stability, and desired accuracy without the need for retraining numerous models for different forecasting horizons. Moreover, we present compelling evidence that models that exploit temporal relationships are better equipped to undertake multi-horizon forecasting tasks.

Fifth, a very simple study of real-time deployment was conducted in [15], while we perform a comprehensive analysis to demonstrate the viability of our solution in real-time industrial environments.

Finally, while the previous work used a fixed temporal window size of 60 s, our work analyzes a wide range of larger temporal windows (150, 300, and 600 s), showing that better performance can be achieved with larger temporal windows for the prediction task. Table 1 presents a summary of the primary findings and contributions of previous studies in the research field that this article addresses.

## 3. Proposed Deep-Learning Architectures

The objective of this section is to explore the main aspects of the time-series forecasting process and to detail the proposed deep-learning models. First, we describe the main techniques used in time-series forecasting and present the mathematical formulations of each technique. Next, we discuss the model selection process, examining the key criteria used to select the forecasting models appropriate to the problem at hand. Finally, we present the proposed new variants of the encoder-only transformer model adapted for the time-series forecasting problem.

### 3.1. Time-Series Forecasting Description

Time-series analysis is a statistical technique that is used to analyze and forecast data points collected over a period of time. In time-series analysis, a key differentiation is drawn between endogenous and exogenous variables. Endogenous variables are those determined by the system being studied, while exogenous variables are determined by external factors that can impact endogenous variables. Specifically, in the realm of time-series forecasting, endogenous variables (*Y*) are data points that are predicted, while exogenous variables $\left(X_{i}\right)$ are not predicted but can be provided as input to the model to improve the accuracy of the forecast.

In the domain of forecasting techniques, one-step ahead forecasting is a widely adopted technique in contemporary forecasting in which the model predicts a single time step ahead of the present time. This method uses observed data and previously predicted steps to produce a forecast that can be used iteratively to generate future predictions indefinitely. However, this technique may not be ideal for long-term forecasting problems as it can result in significant prediction errors due to the accumulation of errors from each intermediate prediction.

From a formal point of view, we can define the one-step ahead forecasting as follows. Let *I* be the unique set of samples in a given time-series dataset. Each sample *i* of the set *I* is associated with a scalar input $X_{i,t}\in R$ and a target $Y_{i,t}\in R$ at each time step $t\in [0,T_{i}]$, where $T_{i}$ is the size of each complete sample *i* (i.e., the length of the time series). The objective of one-step ahead forecasting is to predict the value of the target variable $Y_{i,t\;+\;1}$ in the next time step given the previous predicted steps and the historical data up to time *t*.

$${\hat{Y}}_{i,T}=f\left(Y_{t-K:t},X_{i,t-K:t}\right)$$

where *K* is the size of the past temporal window, and *f* is a function that maps the inputs and history to the predicted output at time t+1. The objective is to identify a suitable function *f* that minimizes the error between the predicted output ${\hat{Y}}_{i,t\;+\;1}$ and the ground truth value $Y_{i,t\;+\;1}$ on a validation set. The size of the past temporal window *K* depends on the problem and, therefore, should be determined empirically.

To generate a forecasting sequence using the one-step ahead forecasting technique, we start with an initial input $X_{i,0}$ and use the model *f* to predict the output $Y_{i,1}$. Then, we use the predicted output as the input for the next time step, i.e., $X_{i,1}=Y_{i,1}$, and repeat the process iteratively to obtain a sequence of predicted outputs $Y_{i,2},Y_{i,3},\ldots ,Y_{i,T_{i}}$. As can be seen, errors in each intermediate prediction can propagate to subsequent predictions, leading to a compounding effect on the overall accuracy of the forecast.

In contrast, fixed-horizon forecasting is designed to predict a specific future time step with optimized accuracy, thus, avoiding the accumulation of errors. However, this technique is limited to predicting only the specific step and cannot be used for earlier or later predictions. Fixed-horizon forecasting is concerned with predicting the target variable $Y_{i,T}$ at a specific future time *T* given the input variables $X_{i,T}$ and the history of the time series up to time *t*, i.e., $\left(X_{i,0},Y_{i,0}\right),\ldots ,\left(X_{i,t},Y_{i,t}\right)$. This can be formalized as follows:

$${\hat{Y}}_{i,T}=f\left(Y_{t-K:t},X_{i,t-K:t}\right)$$

where *T* is the time at which the prediction is made, $Y_{t-K:t}$ represents the past values of the time series up to time *t*, and $X_{i,t-K:t}$ represents the input variables associated with the time series over the same time window. The function *f* maps the historical values and inputs to the predicted value ${\hat{Y}}_{i,T}$ at time *T*. The goal is to find a suitable function *f* that minimizes the prediction error between the predicted value $\hat{Y}i,T$ and the ground truth value Yi,T on a validation set.

Multi-horizon forecasting is an alternative approach that involves predicting a series of future steps. This method aims to provide flexibility in the forecasting process and minimize cumulative errors, although it may not be as accurate as the fixed-horizon method for predicting a single point in time. This forecasting technique involves predicting the target variable for a series of future time steps, i.e., $Y_{i,t\;+\;1},Y_{i,t\;+\;2},\ldots ,Y_{i,T}$. This can be formalized as follows:

$${\hat{Y}}_{i,t\;+\;1:t\;+\;h}=f\left(Y_{t-K:t},X_{i,t-K:t}\right)$$

In the above expression, *h* is the forecast horizon, ${\hat{Y}}_{i,t:t\;+\;h}$ is the predicted sequence of the *h*-step-ahead forecast with respect to the time step *t*, *K* is the size of the past temporal window, and *f* is a function that maps the inputs and history up to time *t* to the predicted sequence at time ${\hat{Y}}_{i,t\;+\;1:t\;+\;h}$. The goal is to find a suitable function $f_{h}$ for each horizon *h* that minimizes the error between the predicted output ${\hat{Y}}_{i,t:t\;+\;h}$ and the ground truth value $Y_{i,t:t\;+\;h}$ on a validation set, typically using a metric that measures the general accuracy of the predictions, such as the mean squared error or the mean absolute error.

However, in the case of DL, a single model can be trained to predict multiple horizons simultaneously, which is known as multi-output multi-input forecasting (MIMO) [17]. In this approach, a deep-learning model is trained to predict the target variable for multiple future time steps, i.e., $Y_{i,t\;+\;1},Y_{i,t\;+\;2},\ldots ,Y_{i,T}$, all at once. This technique allows for more flexibility and accuracy in long-term forecasting as it can capture complex nonlinear relationships between the input and output variables, and the errors do not accumulate over time.

The aim of the present study is to compare the performance of multi-horizon with fixed-horizon forecasting models proposing two new variations of the transformer model assessing their capability for real-time predictions. The following sections provide a comprehensive overview of the models used in this study, including a description of the deep-learning models and the procedures for their training.

### 3.2. Deep-Learning Models

In this section, we focus on the use of deep-learning architectures for multi-horizon forecasting of the AGV deviation. The transformer architecture, initially developed for natural language processing tasks, was selected as a representative of modern deep-learning architectures with demonstrated potential for time-series prediction problems. In this work, two variants of time-series-forecasting models using the transformer encoder-only architecture were proposed: one that applies the attention mechanism on all input data simultaneously and another that only applies the attention mechanism on the time dimension of the input data.

Additionally, the long short-term memory (LSTM) architecture was chosen as a representative of traditional deep-learning architectures that process sequential data due to its ability to learn long-term dependencies. In addition, a one-layer fully connected neural network (FCNN) architecture [18] was selected as a representative of universal regressors to be used as the final stage of our models.

It is important to mention that the FCNN architecture is frequently utilized as the final layer in other deep-learning models, enabling it to offer a final prediction based on the information processed by the previous layers in the deep architecture. In this paper, the LSTM, TRA-TIME, and TRA-FLAT architectures were built adding an FCNN regressor with a single hidden layer as the last stage of the model.

The key difference between the fixed- and multi-horizon models lies in the configuration of this final FCNN layer, where a single output FCNN was utilized for the fixed-horizon models, while a multi-output FCNN was utilized for the multi-horizon models. Furthermore, the multi-horizon architectures were trained to ignore the first 10 predicted steps, as multi-horizon models tend to prioritize optimizing these first steps at the expense of the more crucial later steps.

#### 3.2.1. New Transformer Architectures

The transformer is a state-of-the-art deep-learning architecture based on a sequence-to-sequence model that uses multi-head self-attention layers to effectively process input data [8]. The transformer consists of an encoder and a decoder block, typically used in natural-language-processing (NLP) tasks where the encoder takes in a sentence in one language and the decoder generates a sentence in another language.

The encoder architecture begins with an embedding layer that converts words into numeric vectors, followed by a positional encoding layer that encodes the position of the words in the sentence. The architecture then consists of N sequential blocks starting with a multi-head self-attention layer, followed by a non-linear dense hidden layer. The decoder follows a similar structure with an embedding layer and positional encoding layer for the target language, N sequential blocks starting with multi-head self-attention layers and a multi-head mixed attention layer, and a non-linear dense hidden layer.

The transformer architecture can be adapted to time-series prediction problems. In [10], it was shown that using only the encoder part of the architecture is sufficient for classification and regression problems without the need for the embedding layer. Although the transformer does not have layers that directly process sequential data as RNN or LSTM do, the multi-head self-attention blocks can replicate this behavior by giving different importance to elements in the sequence. This allows the transformer to learn complex sequential behaviors and to be a more general and powerful architecture compared to RNN or LSTM.

In contrast to [8], the architecture shown in [10] had dimension reduction before the trainable positional encoder. Specifically, the dimension reduction block was a linear FCNN with a hidden layer that had fewer outputs than inputs, while the trainable positional encoder block consisted of a linear FCNN with a hidden bias-only layer.

In this study, we propose two variations of the encoder-only transformer model for time-series forecasting [10]:

- Time-Attention Encoder-Only Transformer (TRA-TIME): applies attention only to the time dimension. Unlike in [10], the input data batch is transposed by only swapping the features and the time dimension. This helps the model to learn time-related patterns, such as trends and seasonality. Furthermore, due to the efficiency of this approach in most time-series-related problems, the dimension reduction block used in [10] can be omitted. This omission increases the quality of the information received by the attention layers and the quality of the entire model.
- Flattened-Attention Encoder-Only Transformer (TRA-FLAT): applies attention to all input data. Unlike in [10], before the trainable positional encoder block, the input batch is flattened to a single dimension. This allows the model to generalize and learn data relationships independently of the feature or time. In addition, the model becomes more complex and less efficient. Therefore, the dimension reduction block is still required in most cases.

The first variant (TRA-TIME) is more efficient and effectively exploits time-related patterns compared to the version that attends to all input data. Additionally, the model that focuses solely on the feature dimension, as presented in [10], was not considered due to the fact that half of the feature configurations only had one feature, making the application of attention meaningless. Furthermore, the multi-horizon variants of the TRA-FLAT and TRA-TIME models were obtained by adding a single hidden layer multi-output regression FCNN at the end of the encoder block.

#### 3.2.2. LSTM Models

The long short-term memory (LSTM) is an improved version of the recurrent nNeural network (RNN) architecture, which was proposed to overcome the limitations of RNN [19]. Unlike RNN, LSTM is equipped with an infinite look-back window that allows it to learn the long-term dependencies of the data [20,21]. This architecture operates using a gate-based mechanism that controls the state of each cell to determine whether to store or discard information. These gates are essentially simple neurons that are combined with arithmetic functions and trained together with the rest of the neural cells during optimization. There are three LSTM gates, namely, the forgetting gate, the input gate, and the output gate.

The forgetting gate is responsible for controlling the retention of information from the previous state, while the input gate controls the storage of new information, considering the previous state and the forgetting gate. Finally, the output gate is responsible for controlling the output of the LSTM cell using the internal state updated by the input gate.

The proposed solution attempts to predict in advance the position of a remote controlled AGV that is connected to a virtualized programmable logic controller (PLC) via a 5G network. This specific AGV is a magnetic-guided hybrid differential-tricycle manufactured by ABB with a unique kinematic configuration in which the traction unit operates as a differential robot, and the body follows the movement of a tricycle mobile robot [22].

The kinematics of this AGV are a combination of a trycicle and a differential vehicle. The traction unit moves as a differential robot (1),

(1) $$\dot{\psi }=\frac{\left(v_{r}-v_{l}\right)}{L_{h}}$$

where the relative angle between the traction unit to the body of the AGV (also called the steering angle) is given by ψ. $L_{h}$ is the length of the front axle, and $\left(v_{l},v_{r}\right)$ (m/s and m/s) are the longitudinal speed of the left wheel and right traction wheels.

From the steering angle, ψ, and the total longitudinal velocity, $\left(v_{l}+v_{r}\right)$, the angle of the body of the AGV in the inertial frame, is obtained (2)

(2) $${\dot{\theta }}_{b}=\frac{\left(v_{l}+v_{r}\right)}{2L_{b}}sin\left(\psi \right)$$

where $L_{b}$ is the distance between the axles. The body of the AGV moves like a trycicle, where steering is determined by the traction unit.Thus, considering the velocity, the steering angle and the angle of the body, the evolution of the location of the center of the rear axle, given by $\left(x_{b},y_{b}\right)$, is obtained; Equations (3) and (4)

(3) $${\dot{x}}_{b}=\frac{\left(v_{l}+v_{r}\right)}{2}cos\left(\psi \right)cos\left(\theta_{b}\right)$$

(4) $${\dot{y}}_{b}=\frac{\left(v_{l}+v_{r}\right)}{2}cos\left(\psi \right)sin\left(\theta_{b}\right)$$

Typically, a PLC is adequate to effectively control an AGV that is guided by magnetic tapes. However, when the controller is virtualized and communicates with the AGV through a wireless network, the network infrastructure may introduce new challenges, such as the appearance of errors and delays in the transmission of packets.

In the communication between AGV and PLC, the UDP protocol is used to avoid the processing time overhead and bandwidth consumption introduced by TCP in order to ensure the sequential delivery of packets and error and congestion control. Note that the application protocol between the AGV and the PLC is extremely simple, as the AGV periodically sends a packet to the PLC with the status of its sensors, and the PLC responds with the corresponding guide command to the AGV. Therefore, the error control required in this application protocol is implemented naturally simply by discarding damaged packets at the network level, since eventually, the AGV will resend its status to the PLC, and the PLC will respond with a new guide command to the AGV.

To minimize communication risks, the virtual PLC is located on a (multi-access edge computing) MEC infrastructure, which, in addition to reducing communication latency and errors between the PLC and the AGV, reduces potential security problems that may occur on the supplier’s side outside the AGV factory. Thus, the potential network problems studied are focused on the connection between the MEC and the AGV.

The virtualization of the PLC allows one to reduce hardware-related costs and to control multiple AGVs in a centralized way. Indeed, the MEC and URLLC (ultra-reliable low-latency communication) features of 5G networks enable controlling a large number of AGVs with an unique virtualized PLC over an MEC (outside the AGVs).

The communication between the physical AGV and the virtualized PLC can be summarized in the following steps:

1. The AGV sends status data to the PLC. The most important part for the control application is the deviation of the AGV with respect to the magnetic line of the circuit. This provides an estimate of the position of the AGV.
2. The PLC uses the received AGV position to compute speed references for the wheels to correct its deviation with respect to the magnetic line. This information is sent to the physical AGV.
3. The AGV applies these speed references and returns to step 1 to send its updated status to the PLC.

The latency in this communication is a crucial factor that must be kept within a limited range of tens of milliseconds to guarantee the proper functioning of the system. Failure to meet this requirement could result in the AGV becoming unstable and deviating from its intended path, leading to economic losses due to interrupted production as well as potential health hazards for workers in the vicinity and environmental harm.

We propose a prediction system based on advanced deep-learning techniques to minimize the risks associated with network degradation when the AGV is remote-controlled by a virtualized PLC. This prediction system can be used as part of the AGV control to automatically adjust the AGV speed according to the future quality of network service and generate alerts for the supervisory controller. Furthermore, this prediction service can be deployed in the MEC infrastructure both to intercept the data communicated between the AGV and the PLC and to implement an advanced controller capable of reacting in real time to possible problems in the network.

**Figure 1:**
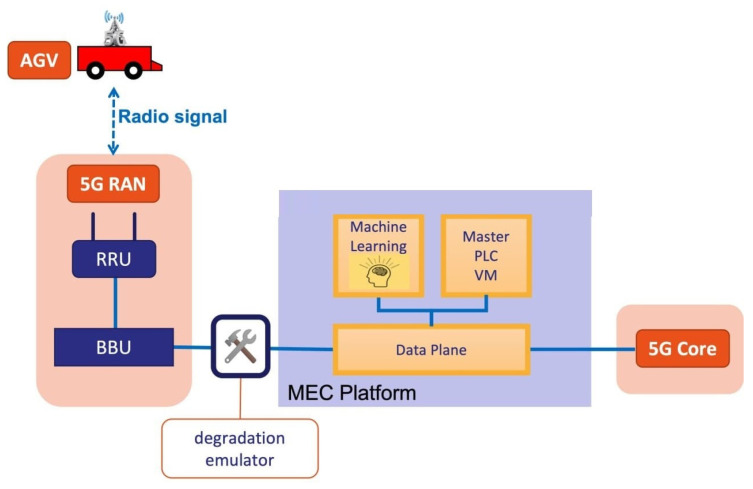
Use case architecture representing the AGV, 5G RAN, 5G MEC, 5G CORE, and ML modules.

The architecture of the proposed use case is shown in Figure 1 and is composed of the AGV, the 5G infrastructure, the MEC platform, the deep-learning model, and the virtualized PLC, both running on the MEC component. However, in order to use a deep-learning model, it must first be trained with a dataset that represents a real situation in which network disturbances can appear. For this purpose, in the experimental scenario, we have extended this use case with a degradation emulator component that emulates the appearance of network disturbances (e.g., delay, jitter, and packet errors) in the communication link between the AGV and the PLC. This component is only necessary in the laboratory phase.

## 4. System Model

**Figure 2:**
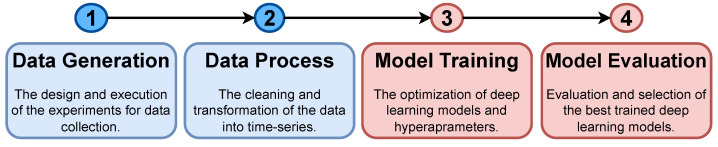
Overview of the steps of the followed method, including: Data Generation (see Section 5.1), Data Processing (see Section 5.2), Model Training (see Section 6), and Model Evaluation (see Section 7).

A well-established methodology, illustrated in Figure 2, was employed to evaluate and compare the selected deep-learning architectures and models. The process consists of the following steps, which are described in detail in subsequent sections: (i) deploying a setup of the AGV that satisfies the use case requirements in the 5G scenario to generate training and testing data (Section 5.1), (ii) cleaning and transforming the collected data into a time series format (Section 5.2), (iii) training various neural network architectures (Section 6), and (iv) evaluating and comparing the trained models to select the best performing models (Section 7).

## 5. Dataset

Deep-learning models, when trained in a supervised manner, require a labeled dataset for learning—preferably a large dataset with a diverse set of scenarios. The process we applied to obtain a dataset for the use case described in Section 4 is as follows:

1. Set up and deploy the components of the use case together with the network degradation generator module.
2. Collect the network packets generated in the communication between an AGV and a PLC under various network degradation scenarios.
3. Clean the collected data and extract the relevant features of the network packets transmitted between the AGV and the PLC.
4. Split the data into different partitions to be used for training the deep-learning models and verifying the proper functioning and generalization of the trained model.

The first two items are detailed in Section 5.1, and the last two are presented in Section 5.2.

### 5.1. Data Collection

A set of labeled data was required to train and test the different forecasting models we selected for our experiments. The data were collected from a set of experiments conducted in a realistic environment where the use case defined in Section 4 was deployed. The use case components (an AGV, the 5G network infrastructure with the MEC module, a PLC virtualized on the MEC infrastructure, and the component that generated the network disturbances) were deployed in 5TONIC, an open laboratory founded by Telefónica focusing in 5G technologies that provides realistic deployments but in a controlled scenario. 5TONIC forms part of a proto Network Digital Twin [23] build on top of the Telefonica Mouseworld Lab [24] and has permanent and temporal infrastructures to set up specific experiments, such as the one we consider in this work.

**Figure 3:**
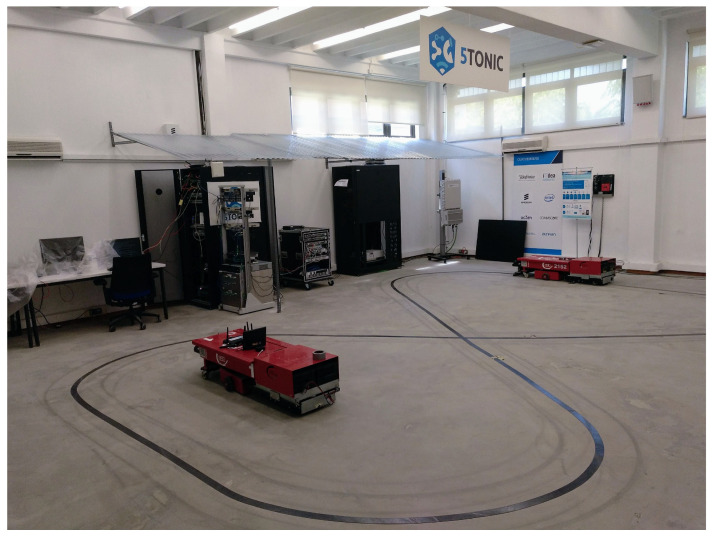
AGV circuit in 5TONIC.

In a room arranged for the scenario setup, a figure-8-shaped circuit was designed (Figure 3). This circuit shape was useful to evaluate the effects of network degradation on the AGV trajectory because, in addition to the possibility that the AGV could leave the circuit in a turn, there was also the possibility that the AGV could make a mistake and change its route at the intersection of the circuit. On top of the MEC infrastructure, a module was deployed to capture the network traffic transmitted between the AGV and the PLC. The traffic was captured in real time using the tcpdump program, which stored it on a disk file in the pcap format. This infrastructure was also used to deploy the real time evaluation of trained forecasting models.

Although 5G networks are known for their robustness and high availability, they can be subject to interference and other disturbances that affect the quality of network service as with any other wireless communication. These network disturbances are reflected in reduced network availability, packet loss in communication, increased latency, and increased jitter. In our experiments, we did not expose the system to real interferences; however, we simulated their effects on network quality by introducing artificially disturbances in the network in the form of packet loss, thereby, increasing the latency and varying jitter.

To this end, a degradation emulator based on the linux tc tool was deployed (Figure 1). The degradation emulator, using a set of statistical patterns, allowed us to vary the network disturbances to simulate situations that could appear in an industrial deployment.

Using the previously defined data capture process, a series of experiment scenarios were designed in which the AGV traversed the circuit several times under different types and degrees of network degradation. The three types of network degradation simulated in the proposed scenarios were: delay, jitter with a pareto-normal distribution (which is considered by experts to be the most realistic jitter degradation type), and the absence of network disturbances.

Other effects were also tested; however, they were not shown to be effective in causing AGV derailment. The intensity of the degradation was defined by the mean value of delay and jitter and by its standard deviation only in the case of jitter. In addition, two types of degradation patterns were used: constant and increasing intensity over time. Constant intensity degradation was applied by maintaining the same average intensity throughout the entire scenario (except for the first and last 30 s) for six rounds of the circuit.

Increasing intensity degradation was defined with an average intensity value at the beginning and a constant average intensity increase every 30 s of the experiment until the AGV deviates and run out of the circuit. Generally, the mean intensity values were defined to be between 100 and 250 ms. Above this value, the experiments were infeasible, and, below this value, the effects were not appreciable.

The standard deviation of the jitter case was defined between 50 and 150 ms. Note that a higher value of standard deviation does not necessarily imply a higher probability of deviation since the AGV has much more risk of deviation due to constant delays than due to momentary delays. Thus, in the case of the increasing degradation, the average degradation intensity started at 100 ms and increased by 50 ms every 30 s until the AGV left the lane.

Each scenario was run at least three times (with the same degradation pattern and intensity), including scenarios without degradation. Although the three runs were run under the same patterns of network disturbances, each run was not exactly the same due to the statistical nature of the introduced disturbances and external factors, such as the presence of dust on the sensors that could indicate the presence of unreal obstacles, and other connections competing for the bandwidth link with the AGV-PLC connection. In addition, as the level of degradation in the network increased, the probability of AGV deviation increased as well. Thus, with the same level of degradation, the AGV may deviate at different time points in the circuit or not deviate at all.

For example, for an average delay value of 250 ms, it was highly likely that the AGV would deviate at least once during its run. With values higher than 350 ms, the AGV was constantly going off, and it became very difficult to perform a complete run of the circuit. Therefore, the scenarios were run at least three times to capture the variation in the environment and the random patterns of the AGV for the same degradation scenario.

Several network degradation scenarios were run, and the capture system stored, for each run, the network packets transmitted between the AGV and the PLC in a different file in the pcap format. The captured network packets were processed using the tstat tool (http://tstat.polito.it/, accessed on 10 February 2023) to extract network statistics, and the application payload of the packets was decoded to extract the AGV guide error on the magnetic circuit.

It is worth noting that the AGV guide error was used as the variable to be forecast by deep-learning models as the value of this variable is directly correlated with the AGV deviation. The communication between the AGV and the PLC was performed using the UDP communication protocol. Therefore, the communication statistics extracted by tstat were reduced to a set of eight features, of which we selected only three of them: packet timestamp, number of packets sent, and number of packets received by the AGV since the beginning of the communication.

The other five features did not provide any useful information as the ratio of bytes sent and received were constant due to the AGV-PLC protocol, and the other four variables were connection status flags. From the decoded information of the packet payload, we only extracted the guide error deviation status of the AGV measured in centimeters to obtain a model with good generalization with respect to other AGVs and to avoid the use of features that may not be included in the communications of other AGVs.

### 5.2. Data Processing

After data collection, the obtained data were converted into time series, after which they were subjected to the necessary data cleaning procedures and subsequently split into separate training and test sets. To facilitate the training of a supervised deep-learning model, a mandatory prerequisite is the collection of data in the appropriate format to facilitate training. In particular, for deep-learning models, it is crucial to feed time series with no missing values.

In order to prepare the data for the model training, the first step taken was to group the data into time series with uniformly separated time steps. Specifically, we regrouped the data every 100 ms based on packet timestamps and used the last observed value for each variable. This approach was chosen because the number of packets and timestamps were variables that only increased over time, and the last value was, thus, the most accurate representation. For the guide error variable, the most recent deviation that was closest in time to the prediction was used. In addition, maintaining the last value of the variables proved to be an efficient method for a real-time problem. The 100 ms time step was experimentally determined to maintain enough information while avoiding excessive redundant data.

Subsequently, null values were filled in using the last observed value. Although rare, null cases may occur if the network is more congested and if more than 100 ms passes between two sequential packets. The last observed value was chosen to fill the null values because of the negligible variation in information between each two sequential steps. In addition, we eliminated the AGV’s stopping moments to recharge the battery from the dataset as these were deemed unnecessary moments to predict, and because the AGV is stopped and cannot deviate during this time, this also had the added benefit of improving the quality of predictions generated by the deep-learning model.

The data were divided into rolling time windows, which is a common practice in the field of time-series forecasting. Specifically, historical rolling time windows were constructed from the time series spanning from time step t−N−1 to t−0, where *t* represents the time step and *N* is the window size. These windows were of fixed size and incorporated both AGV and network features. Multiple window sizes were employed (namely, 15, 30, and 60 s) to determine whether larger window sizes consistently improve the model.

For the fixed-horizon problem, the absolute value of the guide error at a 15 s future step was adopted as the output variable. For the multi-horizon problem, the output time window was defined as the absolute value of the guide error from t+0.1 to t+20 s (200 future steps). To compare the fixed-horizon and multi-horizon predictors, the t+15 s step was selected. It is stated by experts in AGV systems that, due to its inertia, a reaction time of at least 10 s is required to fully stop a loaded AGV that is moving [15]. The remaining 5 s of time were allotted for maneuvering. Moreover, t+15 s represents an intermediate point between the minimum required of t+10 s and the maximum of the multi-horizon prediction problem, t+20 s.

In order to ensure that the model can generalize and make accurate predictions in new experiments, it is essential to separate the data into training and testing sets. In this study, each experiment was performed three times to ensure statistical adequacy. The two first experiments were used for the training set, while the last experiment was used for the testing set. Rather than using the typical method of randomly dividing a single dataset into training and testing sets, a more conservative approach was taken to enable an accurate evaluation of the trained model’s performance using a completely new experiment (i.e., the testing dataset) that was not previously seen during training.

To obtain an accurate deep-learning model, it is necessary to train a large set of models with different hyperparameters in order to identify the optimal set of values. To this end, 10% of the training dataset was randomly extracted to validate the best hyperparameters, and another 10% was extracted to validate the model after each epoch during the training phase. These two new datasets were designated as hyperparameter validation and training validation, respectively. Thus, the final training dataset comprised 80% of the training dataset that was created originally.

## 6. Deep-Learning Model Training

The training of deep-learning models often results in a variety of configurations due to stochastic weighting initialization and variation in optimal hyperparameter settings. To overcome these challenges, we employed the random search method, which facilitates the exploration of numerous hyperparameter and architectural configurations. The selection of the optimal solution was based on a validation dataset. The advantages of this approach are its simplicity and the potential to discover new and unexplored configurations, thereby, decreasing the risk of getting trapped at a local minimum. However, it should be emphasized that, for the final production deployment, it may be prudent to employ a more sophisticated hyperparameter optimization method, such as NSGA-II [25], to obtain the most accurate model.

The random search method was applied based on the hyperparameters and ranges shown in Table 2. In the table, each architecture has two types of hyperparameters: structure and robustness. The structure hyperparameters define the structure of the architecture, while the robustness hyperparameters reduce the possibility of overfitting. For the long short-term memory (LSTM) architecture, the structure hyperparameters include the number of layers and the number of LSTM neurons per layer.

The structure hyperparameters for the transformers include the reduction size of the fully connected neural network (FCNN) layer located at the beginning of the architecture. This layer is only required for the transformer that places attention on all input data (TRA-FLAT) to reduce them to the required dimensions of the first multi-head attention block. However, the transformer that only places attention on the time dimension (TRA-TIME) does not require the reduction size FCNN layer.

Additionally, the number of blocks, heads, and intermediate neurons per block defines the multi-head attention blocks of the transformer architecture. For the FCNN architecture, the number of neurons in its single hidden layer defines its structure hyperparameters. Finally, some of the robustness hyperparameters, such as dropout, L2 regularization, and batch normalization, may be shared among the architectures if necessary.

In each iteration of the random search, a set of hyperparameter configurations was generated for each type of deep-learning model (LSTM, TRA-FLAT, and TRA-TIME) and for each input time-window size (15, 30, and 60 s). The hyperparameter configurations were then used to train each deep-learning model with the corresponding time-window configuration. To reduce the risk of overfitting, each model was evaluated against a validation dataset after each training epoch during the training process.

The best hyperparameter configuration was selected for each deep architecture and time-window size using the hyperparameter validation dataset. Finally, the selected models were tested against the test dataset for the final evaluation and selection of the best deep-learning model and the best time-window size. It is worth mentioning that the test dataset was collected from the last execution of each scenario and used to evaluate the models’ generalization capabilities for future scenarios.

The assessment of deep-learning models will rely on the subsequent metrics: the mean absolute error (MAE), symmetric mean absolute percentage error (SMAPE), and prediction speed. The MAE serves as a measure of the model’s predictive error in absolute terms and is used as a comparison metric to identify the best model. The SMAPE provides a comprehensive indicator of the error committed relative to the mean prediction value, and in industrial settings, where a SMAPE below 50% is deemed accurate [26]. The prediction speed measures the model’s feasibility for real-time inference, and, in our context, it must exceed 10 predictions per second, as the data is going to be sampled every 100 ms (in the real-time solution).

It is evident that each metric will provide different insights into the trained model. The MAE will assist in identifying the best model, whereas the SMAPE and prediction speed will reveal the model’s practicality. In the experiments section, we evaluate and discuss the models using selected hyperparameter configurations based on the second validation dataset and test dataset.

## 7. Experiments

To evaluate the effectiveness of the deep-learning models, a series of experiments were conducted. The selected deep-learning architectures were LSTM and the two variants of encoder-only transformers proposed in this work: (i) TRA-FLAT that attends to all input features and (ii) TRA-TIME that only attends to the time dimension of the input features. These models were modified to predict with either a fixed-horizon (15 s ahead) or a multi-horizon (200 steps ahead, ranging from 0.1 to 20 s), resulting in either one or multiple outputs.

The input data were varied in terms of time-window sizes of 15, 30, and 60 s, and the impact of incorporating statistical network information into the historical guide error values was also evaluated. All 36 architecture configurations and input data were trained with at least 20 random hyperparameter configurations as explained in Section 6. In total, 720 deep-learning models were evaluated using the test dataset. The models were trained and tested on an off-the-shelf computer equipped with a GeForce RTX 3090 Ti GPU, a 12th Generation Intel(R) Core(TM) i9-12900K CPU, and 128 GiB of RAM.

The results of these experiments are analyzed in the following subsections. First, we compare the precision of the 15th second step prediction of a multi-horizon model with that of a fixed-horizon model, and show that the best multi-horizon model can predict with a similar level of precision. Second, we compare and discuss the best performing multi-horizon models and find that architectures that consider time relations in their input data, such as LSTM and time-attention transformers, perform better than the models that do not attempt to exploit the temporal topology of the input data. Finally, we provide guidance on how to select and deploy the best deep-learning models and demonstrate that all models can be used in a industry real-time scenario.

### 7.1. Fixed-Horizon Forecasting

In the realm of predictive modeling, it can be supposed that a fixed-horizon model, having been tuned to optimally forecast a single time step, would exhibit greater accuracy in predicting that specific time step, whereas the multi-horizon model, being calibrated to minimize the average prediction error of numerous steps, would manifest a lower accuracy in predicting that same time step.

Nevertheless, given that predictions that are closer to the current time tend to be the most accurate, the multi-horizon model allows for dynamic adjustment of the forecasting horizon to accommodate accuracy or predict further into the future, providing the system or operator with the option to select the optimal step based on the workload of the AGV fleet and the required accuracy. On the contrary, using the fixed-horizon forecasting approach to achieve the equivalent versatility of a multi-horizon approach would necessitate the training and validation of a multitude of fixed-horizon models, which could be extremely resource intensive.

To evaluate the difference between fixed-horizon models and multi-horizon models for the same future step and to determine if fixed-horizon models were always better, an analysis was conducted of the comparison between fixed- and multi-horizon models for the 15 s step. The fixed-horizon model was optimized for predicting 15 s ahead, while the multi-horizon model was trained to forecast a range of 200 steps, from t + 0.1 s to t + 20 s ahead.

**Figure 4:**
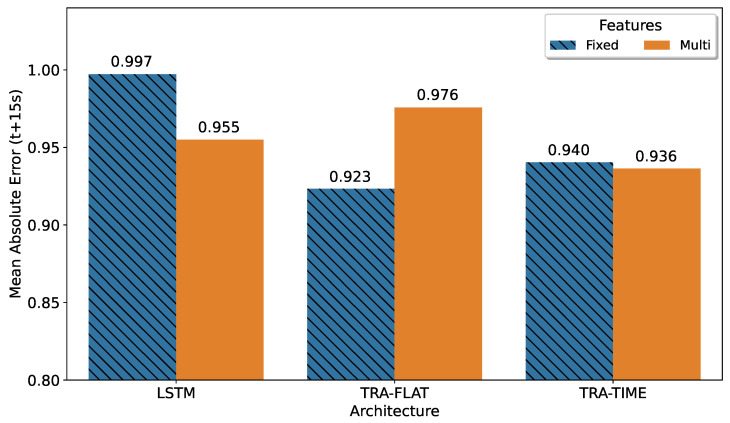
A comparison of the performance of fixed vs. multi horizon models is presented in terms of mean absolute error (MAE) for the prediction of the t + 15 s step. The results presented are based solely on the guide error as the input feature and a time-window size of 60 s, which was determined to be the best configuration. The highest level of precision was attained by the transformer that considered all features (TRA-FLAT) in its fixed horizon configuration, resulting in a mean absolute error (MAE) of 0.923. On the other hand, the best multi horizon model was the transformer that focused only on the time dimension (TRA-TIME), with a slightly lower MAE of 0.936, which only deviates by 1% from the best model—yet offers greater robustness and flexibility.

To evaluate the prediction error, we selected the 150th step from the multi-horizon model’s prediction. The best models for each architecture were selected based on the smallest mean absolute error value. The results, shown in Figure 4, indicated that the best result corresponded to a fixed-horizon model, as expected. The transformer that attends all features (TRA-FLAT) achieved its optimal results in the fixed-horizon approach, while the other models performed best in the multi-horizon format.

This result can be explained because the TRA-FLAT architecture does not consider any topological relationships in the temporal structure of the input data, and therefore its training process focuses exclusively on minimizing the error between the forecast points and real values. For fixed-horizon TRA-FLAT models, only the forecast error at a single point in the future (t + 15) was minimized during training. However, in multi-horizon TRA-FLAT models, the forecast error for all 200 points in the interval $\left[t_{0.1}\ldots t_{200}\right]$ of the multi-horizon output must be minimized simultaneously during training, resulting in less accurate multi-horizon TRA-FLAT models than their fixed-horizon counterparts.

Conversely, architectures that do have the ability to extract temporal information from input data (TRA-TIME and LSTM) achieved better results in a specific point (t + 15) when configured in multi-horizon mode compared to their counterparts in fixed-horizon mode. This suggests that these models are able to leverage the temporal topology learned from past values to improve the accuracy of multi-horizon predictions, particularly at the point of interest (t + 15) and potentially throughout the entire prediction horizon.

It is worth noting that the difference between the best fixed-horizon solution and the multi-horizon transformer that only attends the time dimension (TRA-TIME) was less than 1%, indicating that the versatile multi-horizon model could be utilized with minimal loss in accuracy in the same scenario than the best fixed-horizon solution. This result shows that it is possible to utilize the flexibility of the multi-horizon model without sacrificing accuracy when compared to fixed-horizon models.

### 7.2. Multi-Horizon Forecasting

The multi-horizon models offer a versatile solution for the system or technician to choose the most appropriate forecasting step based on the AGV load and the desired precision using a single model. Multi-horizon model predictions that are closer to the present tend to be more accurate than distant predictions. Additionally, it is expected that sequence-based architectures will perform better in multi-horizon time series problems as they can take advantage of the temporal topological structure of past inputs to generate predictions ahead of time.

**Figure 5:**
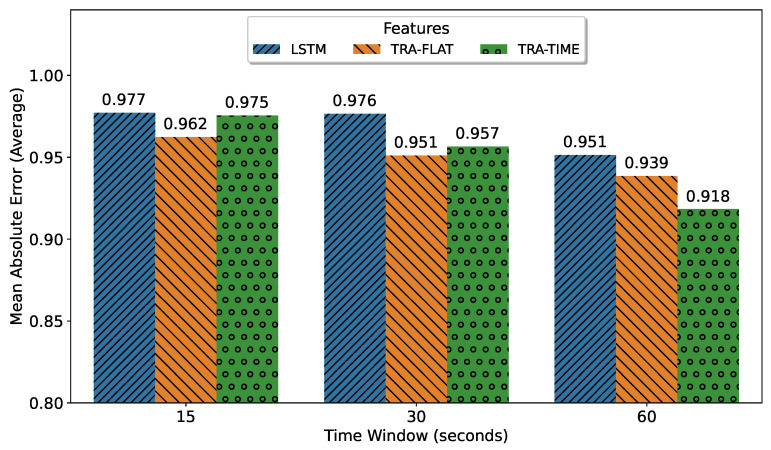
Comparison of the most efficient multi-horizon architectures and input time-window sizes in terms of the average mean absolute error (MAE) across all predicted steps. The performance of all architectures improved as the input time-window size increased while using only the guide error as the input feature. The best performing architecture, with an MAE of 0.918, was the transformer with attention to time features only (TRA-TIME) using a 60 s input time window.

The objective of this subsection was to identify the optimal deep-learning architecture for multi-horizon forecasting based on its precision degradation over successive predicted time steps. As illustrated in Figure 5, the encoder-only transformer with temporal attention (TRA-TIME), utilizing a 60 s input time window, remains the superior deep-learning architecture and model for multi-horizon forecasting. This model was previously identified in Figure 4 as the best multi-horizon model for a single t + 15 s step but was evaluated here based on all predicted steps. Furthermore, it was observed that increasing the amount of input data led to better performance for all architectures.

**Figure 6:**
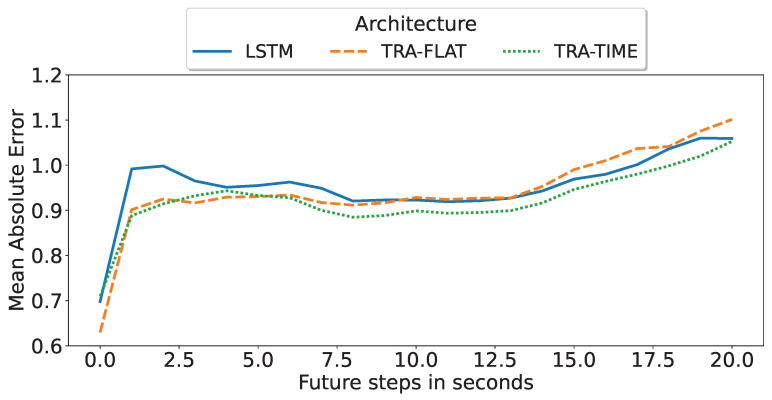
Comparison of MAE values across steps in the forecasting horizon. The horizontal axis represents the second of each step and the vertical axis represents the MAE. The best performing architecture for multi-horizon forecasting was TRA-TIME with an average MAE of 0.918, followed by TRA-FLAT with an average MAE of 0.939, and finally LSTM with an average MAE of 0.951. The figure clearly demonstrates that the LSTM model outperformed the TRA-FLAT after the tenth prediction second, which is significant as a fully loaded AGV requires at least 10 s to stop.

To further analyze the results, we selected the best model for each architecture based on the mean value of the mean absolute error of all predicted steps as shown in Figure 5. Figure 6 compares the performance of each of the best multi-horizon models in predicting each step. The best model was found to be the encoder-only transformer with attention in time (TRA-TIME), followed by the transformer with attention in all features (TRA-FLAT), and finally the LSTM model. However, we observed that the TRA-FLAT model performed better than the LSTM model only before the tenth second of the forecast, while the LSTM model performed better after that time. Recall that a loaded AGV requires at least 10 s for a full stop, so predictions after the tenth second are more important than those before the tenth second.

In conclusion, after a comprehensive evaluation of the multi-horizon models, the encoder-only transformer with temporal attention (TRA-TIME) was identified as the optimal model, followed by the LSTM, with the TRA-FLAT performing the worst. The models’ precision was found to increase as the input time-window size increased. Furthermore, although the TRA-FLAT model exhibited superior performance on average compared to the LSTM model, the LSTM model outperformed the former for critical predictions (beyond the tenth second). Thus, we demonstrated that the time-based architectures outperformed other models for the multi-horizon prediction problem investigated in this study.

### 7.3. Model Deployment

Prior to the deployment of deep-learning models in real-world settings, various verification steps are undertaken to reduce the risk of overfitting, ensure acceptable precision in an industrial context, and ensure that the model can predict at the same rate or faster than the rate of new measurements being sampled. This section presents the outcomes of the verifications and final observations on the selection of the best model.

The initial verification step aims to mitigate the risk of overfitting by assessing the generalization capabilities of the best deep-learning models using a separate dataset. As explained in Section 5, a series of experiments was conducted with a minimum of three repetitions with the last repetition reserved for the test dataset. Since each repetition was conducted in a different period of time, the distinctions between repetitions were considered sufficient to ensure that the test dataset provided generalization of the trained models. Notably, the assessment metrics presented in the preceding subsections were computed using the test dataset, thus, providing a significant reduction in the likelihood of overfitting.

The second verification measure aimed to ensure that the model is suitable for industrial applications, and to achieve this objective, the symmetric mean absolute percentage error (SMAPE) must be below 50%. The SMAPE represents the percentage error deviation from the mean prediction value. As shown in Table 3, all models had a SMAPE value below 50% with the best models displaying a SMAPE of 26%, confirming that the models were sufficiently accurate for industrial applications.

The last verification step was aimed at confirming that the model is capable of keeping up with the data sampling system in real-time. In particular, the sampling system captures packets from a 5G network with a speed of up to 20 Gbps; however, the transformation of the problem into a time series limits the sampling time to 10 summarized packets per second. The deep-learning model is considered suitable for real-time deployment if it can make predictions at a rate of at least 10 predictions per second. Based on the summary results presented in Table 3, all models were at least ten-times faster than the required prediction rate with the slowest model achieving a prediction rate of 124 predictions per second.

It can be concluded that the models presented in Table 3 meet the minimum requirements for deployment in a real-time industrial system. All the models presented in the table are suitable for real-time deployment as they are faster than 10 predictions per second (the data collection speed) and have a SMAPE less than 50% (sufficiently precise for industrial applications).

The best model for the multi-horizon problem is the encoder-only transformer with attention on time features (TRA-TIME) using a 60 s input time window without network features. Furthermore, the best model for the fixed-horizon problem with a 15 s step is the encoder-only transformer with attention on all features (TRA-FLAT) also using a 60 s input time window without network features.

In the case of small time windows of 15 s, TRA-FLAT remains the top-performing model for a fixed-horizon problem. However, for a multi-horizon problem, using TRA-TIME with a 15 s time window yielded better results compared to LSTM and TRA-FLAT when predicting values beyond 10 s (Figure 6). Recall that a forecast for a loaded AGV must predict 10 s or more into the future to ensure that the AGV can be stopped in time. As a result, the most important forecasts in the multi-horizon scenario are those that go beyond 10 s.

It is worth noting that the results of the table suggest that the use of network features is most useful for small time windows and only with fixed-horizon models. On the contrary, it can be observed that the multi-horizon problem prioritizes extracting and learning time-related error patterns, which may not necessarily correspond with the behavior of network disturbances. As expected, an increase in the number of neurons, input window size, or input features leads to a slower prediction speed. It should be noted that the multi-horizon model outputs 200 predictions, while the fixed-horizon model only outputs one.

It is important to mention that the network disturbances observed in our experiments are similar to those encountered in 5G networks deployed in real-world settings, and the physical components of the AGV (such as the guide error sensor) are not likely to experience significant degradation over extended periods of time (i.e., several months) that would alter their physical response and affect the prediction performance.

Based on these observations, we anticipate that the deep-learning models employed in this use case would not require frequent retraining (such as every few days or weeks) and can continue to be effective for an extended period. It is only when several months have passed or when significant environmental changes occur that retraining the model may become necessary to address data drift issues.

During the conducted experiments, the training of the selected deep neural network models had an average duration of 30 min, the 90th percentile completed within 1 h, and the maximum training duration of a model was 7 h due to its large size and lengthy convergence process. Therefore, assuming that the training times obtained are in the range of hours, it can be assured in our use case that retraining can be initiated as soon as a data drift problem is identified, and a new model can be deployed in a reasonably short period of time. To expedite the retraining process, incremental training techniques, such as Transfer Learning, could be beneficial.

## 8. Discussion of Results

This section aims to provide a comprehensive overview of the primary outcomes obtained from the experiments performed, which are presented subsequently.

- Comparison between Fixed-Horizon Models and Multi-Horizon Models: The results of the experiments conducted showed that the best performance was observed for fixed-horizon models, which was in line with prior expectations. However, the difference between the optimal fixed-horizon solution and the optimal multi-horizon solution was found to be less than 1%, and its accuracy slowly decayed as the prediction horizon expands into the future. This indicates that a flexible model with multi-horizons can be used with minimal loss of precision. This outcome holds substantial significance as multi-horizon models offer the system operator or technician the capability to choose dynamically the most suitable forecasting step in accordance with the AGV workload, network stability, and desired accuracy without the need for training and validating a large number of new models for each different forecasting horizon point.
- Performance Comparison between the Different Evaluated Architectures: The experiments indicated that one of our variations of the encoder-only transformer was the best performing model when trained as a fixed-horizon model. In particular, the encoder-only transformer with attention to all features (TRA-FLAT) and using a 60 s input time window without network input features was identified as the best model for the fixed-horizon problem. Conversely, the best model for the multi-horizon problem was found to be the encoder-only transformer with attention to time features (TRA-TIME) utilizing a 60 s input time window that comprised only the AGV guide error variable. Furthermore, the results obtained suggest that network input features are only useful for models operating with small time windows and in the context of fixed-horizon models as multi-horizon models do not seem to exploit this exogenous variable.
- Importance of the Temporal Dimension in Multi-Horizon Forecasting Scenarios: We observed that architectures that exploit the temporal relationships in their inputs, such as LSTMs and encoder-only transformer models with attention mechanisms for the temporal dimension (TRA-TIME), were more effective in multi-horizon forecasting scenarios than (i) their counterparts for fixed-horizon scenarios and (ii) other multi-horizon models that do not consider a temporal topology in their inputs. This result highlights the importance of considering architectures that are well-suited to learn patterns from the temporal dimension when forecasting in multi-horizon scenarios.
- Influence of Input Time Window Size: The results indicated that increasing the size of the input time window was positively correlated with an increase in the accuracy of the models. This relationship was found to be the most pronounced for multi-horizon models, which are known to benefit from the consideration of time relationships.
- Deployment in Real-World Scenarios: The outcomes of the model deployment evaluation were found to be positive and highly supportive of the feasibility of using the models in real-time industrial scenarios. The models were evaluated using the symmetric mean absolute percentage error (SMAPE) assessment and all models exhibited a SMAPE below 50% (sufficiently precise for industrial applications) with the most optimal models having an SMAPE of 26%. Additionally, the models were tested for their ability to manage real-time data sampling, where they had to produce at least 10 predictions per second. The results showed that all models were at least ten-times faster than the minimum requirement with the lowest performing model still capable of producing 124 predictions per second. These results indicate the suitability of the models for real-time industrial use.

## 9. Conclusions and Future Work

In this section, we conclude by summarizing the main findings derived from the results obtained in this work and present interesting future work to explore.

### 9.1. Conclusions

This research aimed to leverage the capabilities of transformers, a state-of-the-art deep-learning (DL) technique, to anticipate the long-term failure of a remotely controlled automated guided vehicle (AGV) when its connection is degraded due to the appearance of network perturbations. To achieve this goal, a virtualized programmable logic controller (PLC) was deployed within a multi-access edge-computing (MEC) infrastructure and connected to an AGV via a 5G network to ensure that minimal latencies were achieved, thus, enabling real-time remote control of the AGV. To train and validate the predictive models, a large dataset was collected through a multitude of experiments conducted in the 5TONIC laboratory. These experiments consisted of simulating various degradation scenarios of the network connection between the AGV and the PLC, inducing a series of network disturbances, such as delays and jitter, in order to mimic realistic conditions.

In this context, we explored the difference between fixed-horizon and multi-horizon prediction models to anticipate future AGV deviations, considering the different state-of-the-art deep-learning models available for time-series forecasting. In particular, we employed two types of deep-learning architectures, namely LSTMs and transformers, for which we proposed, for the first time, two variations of the encoder-only transformer architecture for multi-horizon prediction: one that attended to all features by flattening the input dimensions into one (TRA-FLAT) and one that only attended to time features (TRA-TIME).

The models were tested with either a fixed-horizon (15 s ahead) or a multi-horizon (200 steps ahead, ranging from 0.1 to 20 s) and with varying input data in terms of time-window sizes (15, 30, and 60 s). The impact of incorporating statistical network information obtained from the aggregation of network packets transmitted between the AGV and the PLC to the past values of the predicted variable extracted from the AGV sensor data (guide error) was also evaluated.

Overall, the results of the experiments support the feasibility of leveraging advanced transformer-based models to forecast long-term trajectory deviations in a modern AGV system connected to a virtualized PLC by means of a 5G MEC platform. The results obtained in this work demonstrate the undeniable superiority of multi-horizon forecasting models over their fixed-horizon counterparts as well as the importance of selecting architectures that are well-adapted to learning patterns of the temporal dimension when forecasting in multi-horizon scenarios. Furthermore, the proposed models can perform inference within the required time constraints for real-time decision making.

### 9.2. Future Work

The present study can be extended in multiple ways. First, it is advisable to investigate alternative deep-learning architectures, such as bidirectional long short-term memory models, in an attempt to optimize prediction accuracy. Secondly, researchers can design an AGV control system that adjusts its speed based on the predictions from the forecasting model. Thirdly, future work can gather data from multiple AGVs and assess the effectiveness of using a single model for all the vehicles. Fourthly, optimal strategies for identifying data anomalies that might occur during the manufacturing process should be evaluated in order to automatically initiate model retraining using data accumulated during the AGV fleet’s operational procedures.

Finally, it has been observed that larger windows produce more favorable results, and there is a remarkable positive correlation between the size of the temporal window and the accuracy of the predictions. Therefore, future experiments could be made that extend the current experimentation to incorporate larger windows to achieve potential gains in performance.

## Figures and Tables

**Table 1:** Comprehensive analysis of studies related to the current research topic.

Contribution	Yaovaja et al. [16]	Vakaruk et al. [15]	Our Work
**Type of Models**	Kinematic	Fixed-Horizon Prediction	Multi-Horizon and Fixed-Horizon Prediction
**Use of AI Models**	No	Traditional ML Architectures	State-of-the-Art DL Architectures
**Collision Avoidance (Humans/Obstacles)**	No	Yes	Yes
**Correction of Trajectory Deviation**	Yes	Yes	Yes
**Trajectory Deviation Anticipation**	No	Yes	Yes
**Use of Industrial-Grade Components in Experiments**	No	Yes	Yes
**Use of State-of-the-Art Techniques**	No	Yes	Yes
**Comparison of Fixed-Horizon and Multi-Horizon Prediction Models**	No	No	Yes
**Analysis of the Response Time of the Control Algorithm**	No	No	Yes
**Verification of the Deployment Feasibility**	No	Limited	Yes
**Evaluation under Realistic Conditions in a Real Industrial Environment**	No	Yes	Yes

**Table 2:** Hyperparameter settings for the transformer, LSTM, and FCNN models. The table displays the architecture type in the first column, the hyperparameter name in the second column, the type of hyperparameter value in the third column, and the range of hyperparameter values in the fourth column. The FCNN model is represented as the regression component at the end of the transformer and LSTM models. (*) The reduction size hyperparameter is exclusive to the transformer architecture that focuses on all features (TRA-FLAT).

Architecture	Hyperparameter	Value Type	Value Range
Transformer	Reduction Size *	Integer	[64, 4096]
	Number of Blocks	Integer	[1, 8]
	Number of Heads	Integer	[1, 10]
	Number of Block Output Neurons	Integer	[128, 8192]
	Dropout	Float	[$1\times 10^{-5}$, 0.5]
LSTM	Number of Layers	Integer	[1, 5]
	Number of Neurons per Layer	Integer	[16, 128]
	Dropout	Float	[$1\times 10^{-5}$, 0.5]
	Batch Normalization	Boolean	False/True
	L2 Penalty Term	Float	[$1\times 10^{-5}$, 1.0]
FCNN	Number of Neurons per Layer	Integer	[256, 4096]
	Dropout	Float	[$1\times 10^{-5}$, 0.5]

**Table 3:** The best deep-learning architectures. Deep-learning architectures, the size of the input time window in seconds (TW), the type of model (fixed or multi-horizon), the input features (guide error only or with network), the mean absolute error (MAE), the symmetric mean absolute percentage error (SMAPE) for a 15 s step, the average value of the mean absolute errors for all steps (only for multi-horizon models), and the number of predictions per second are presented. The deep-learning architecture can be either a long short-term memory (LSTM) network defined by the number of layers (L-2) and number of LSTM neurons in each layer (×119), followed by the number of output neurons (F-499), or a transformer with flat attention (TRA-FLAT) defined by the number of output neurons in the reduction layer (R-398), the number of attention blocks (B-4) and number of neurons in each block (×2947), the number of heads in each block (H-4), and the number of output neurons (F-2796). The transformer with time attention (TRA-TIME) is defined in a similar manner as the TRA-FLAT but without the reduction layer. All the models displayed are suitable for real-time deployment, as they are faster than 10 predictions per second (the data collection speed) and have a SMAPE less than 50% (sufficiently precise for industrial applications). The smallest MAE value for the 15 s step of each architecture is highlighted in bold.

				MAE	SMAPE	MAE	Predictions
Architecture	TW	Horizon	Features	15 s	15 s	Average	/Second
LSTM (L-2×119, F-499)	15 s	Fixed	GE + Net	1.052	29%	N/A	411.311
LSTM (L-2×112, F-446)	15 s	Multi	GE	1.011	27%	0.977	480.215
LSTM (L-2×121, F-609)	30 s	Fixed	GE + Net	1.058	28%	N/A	381.997
LSTM (L-2×124, F-821)	30 s	Multi	GE	1.000	27%	0.976	396.051
LSTM (L-2×126, F-503)	60 s	Fixed	GE	0.997	26%	N/A	385.698
**LSTM (L-2×117, F-247)**	**60 s**	**Multi**	**GE**	**0.955**	**26%**	**0.951**	**368.333**
TRA-FLAT (R-398, B-4×2947, H-4, F-2796)	15 s	Fixed	GE	0.992	28%	N/A	176.139
TRA-FLAT (R-344, B-4×5948, H-5, F-3083)	15 s	Multi	GE	1.010	28%	0.962	156.427
TRA-FLAT (R-936, B-2×6292, H-3, F-1759)	30 s	Fixed	GE + Net	0.962	27%	N/A	220.363
TRA-FLAT (R-490, B-5×3353, H-3, F-1441)	30 s	Multi	GE	1.009	28%	0.951	175.153
**TRA-FLAT (R-378, B-4×4917, H-3, F-2671)**	**60 s**	**Fixed**	**GE**	**0.923**	**26%**	**N/A**	**187.143**
TRA-FLAT (R-658, B-6×3240, H-3, F-1282)	60 s	Multi	GE	0.976	27%	0.939	124.321
TRA-TIME (B-2×5581, H-4, F-832)	15 s	Fixed	GE	0.987	27%	N/A	279.559
TRA-TIME (B-3×7637, H-4, F-1959)	15 s	Multi	GE	1.015	28%	0.975	215.980
TRA-TIME (B-2×5846, H-4, F-989)	30 s	Fixed	GE	0.968	26%	N/A	277.005
TRA-TIME (B-4×7685, H-4, F-1748)	30 s	Multi	GE	0.994	28%	0.957	182.018
TRA-TIME (B-1×6937, H-4, F-1275)	60 s	Fixed	GE	0.940	26%	N/A	448.991
**TRA-TIME (B-2×8014, H-5, F-753)**	**60 s**	**Multi**	**GE**	**0.936**	**26%**	**0.918**	**258.026**

## Data Availability

The data that support the findings of this study are available from Telefonica and ASTI Mobile Robotics but restrictions apply to the availability of these data, which were used under license for the current study, and so are not publicly available. The data are, however, available from the authors upon reasonable request and with permission of Telefonica and ASTI Mobile Robotics.
